# A mindfulness-based intervention for breast cancer patients with cognitive impairment after chemotherapy: study protocol of a three-group randomized controlled trial

**DOI:** 10.1186/s13063-020-4204-8

**Published:** 2020-03-23

**Authors:** Katleen Van der Gucht, Michelle Melis, Soumaya Ahmadoun, Anneleen Gebruers, Ann Smeets, Mathieu Vandenbulcke, Hans Wildiers, Patrick Neven, Peter Kuppens, Filip Raes, Stefan Sunaert, Sabine Deprez

**Affiliations:** 1 Leuven Mindfulness Centre, Faculty of Psychology and Educational Sciences, University of Leuven, Tiensestraat 102, 3000 Leuven, Belgium; 2 Department of Imaging and Pathology, KU Leuven, Herestraat 49, 3000 Leuven, Belgium; 3 Multidisciplinary Breast Centre, University Hospitals Leuven, Herestraat 49, 3000 Leuven, Belgium; 4 Department of Oncology, KU Leuven, Leuven, Belgium; 5 Department of Surgical Oncology, University Hospitals Leuven, Herestraat 49, 3000 Leuven, Belgium; 6 Department of Neurosciences, KU Leuven, Leuven, Belgium; 7 Department of Psychiatry, University Hospitals Leuven, Herestraat 49, 3000 Leuven, Belgium; 8 Department of General Medical Oncology, University Hospitals Leuven, Herestraat 49, 3000 Leuven, Belgium; 9 Department of Gynaecology and Obstetrics, University Hospitals Leuven, Herestraat 49, 3000 Leuven, Belgium; 10 Department of Radiology, University Hospitals Leuven, Herestraat 49, 3000 Leuven, Belgium

**Keywords:** Cognitive impairment, Breast cancer, Mindfulness, Randomized controlled trial, Brain imaging

## Abstract

**Background:**

Mindfulness has been applied to improve cancer care by enhancing psychological well-being. However, little is known about its impact on cognitive impairment experienced by cancer patients after chemotherapy. Mindfulness may be relevant in tackling cognitive impairment by decreasing emotional distress and fatigue, by decreasing inflammation, and by strengthening functional brain connectivity. The aim of the present study protocol is to evaluate the efficacy and mechanisms of a mindfulness-based intervention to reduce cognitive impairment in breast cancer patients after chemotherapy.

**Methods/design:**

The present study is a three-arm, parallel-group, randomized controlled trial with assessments at baseline, 1 to 3 weeks after the intervention and at 3 months’ follow-up. One hundred and twenty breast cancer patients who ended treatment a minimum of 6 months and a maximum of 5 years before, and who have cognitive complaints, will be enrolled. They will be randomized into one of the following three study arms: (1) a mindfulness-based intervention group (*n* = 40), (2) an active control condition based on physical training (*n* = 40), or (3) a treatment as usual (TAU) control group (*n* = 40). Both the mindfulness-based intervention and the active control condition consist of four group sessions (3 h for the mindfulness condition and 2 h for the physical training) spread over 8 weeks. The primary outcomes will be cognitive symptoms as measured by the Cognitive Failure Questionnaire and changes in functional brain connectivity in the attention network. Secondary outcomes will be (1) levels of emotional distress, fatigue, mindfulness, quality of life; (2) neurocognitive tests; (3) structural and functional brain changes using MR imaging and (4) measures of inflammation.

**Discussion:**

The study will examine the impact of a mindfulness-based intervention on cognitive impairment in breast cancer patients. If the findings of this study confirm the effectiveness of a mindfulness-based program to reduce cognitive impairment, it will be possible to improve quality of life for ex-cancer patients. We will inform health care providers about the potential use of a mindfulness-based intervention as a non-pharmaceutical, low-threshold mental health intervention to improve cognitive impairment after cancer.

**Trial registration:**

ClinicalTrials.gov, ID: NCT03736460. Retrospectively registered on 8 November 2018.

## Background

The prevalence of cognitive impairment in cancer patients has become an important area of research. There is increasing evidence that chemotherapy treatment for breast cancer can have both acute and long-term effects on cognitive functioning [1, 2]. Women treated for breast cancer regularly self-report problems with cognitive processes involving memory, attention and executive functioning [3]. These cognitive deficits can be a worrying side effect of cancer and its treatment and can have a serious impact on quality of life and productivity at work, bringing undesirable direct and indirect costs to patients and society. Researchers cite the incidence of post-treatment cognitive problems as ranging from 18 to 78% [3]. The course and duration of treatment-related cognitive dysfunction is largely unknown. Frequencies are higher shortly following the completion of treatment [2, 4]. While one group of women shows (partial) recovery at 1 year post chemotherapy [1], others still experience cognitive dysfunction 10 to 20 years after treatment [5, 6].

Different candidate mechanisms for cancer therapy-related cognitive changes have been proposed and include direct neurotoxic effects of chemotherapeutic agents, indirect immune-mediated inflammatory processes, induced hormonal changes, and genetic predisposition [7]. Furthermore, cancer-related symptoms, such as fatigue, anxiety, depression and stress, can have an additional impact on cognitive performance [8]. Studies also show that cognitive impairment following chemotherapy is associated with structural and/or functional changes in the brain, more specific in the white matter microstructure which could be linked with decreases in cognitive performance [9, 10]. Changes in brain activation were observed both in the attention network during the execution of active tasks (e.g., multi-tasking) [11] as well as during rest in the default-mode network [12].

There is a great need for therapeutic interventions that can reduce cognitive symptoms after cancer treatment. So far, only a limited number of potential interventions targeting cancer-related cognitive impairment have been studied [13]. The most promising interventions so far appear to be cognitive training and physical activity [13, 14]. However, due to a lack of active control groups and the consideration of biological outcomes, it remains unclear to what extent these interventions are effective.

Recently, more attention has been given to mindfulness-based interventions (MBIs) as a potential candidate-intervention to reduce cognitive symptoms in cancer patients. A MBI is an evidence-based intervention teaching participants to pay attention to whatever arises, in the here and now, in a compassionate and non-judgmental manner [15]. During MBI participants develop skills in their capacity to become non-judgmentally aware of thoughts, feelings and sensations, and increase their capacity to replace automatic, habitual reactions with more conscious responses. This training in attention and fostering awareness can be done through a combination of formal mindfulness meditation exercises and informal practice by being more attentive and engaged in daily life.

A MBI may have an impact on cognitive impairment through multiple pathways: (1) MBIs alleviate symptoms of emotional distress and fatigue, factors known to have an impact on cognitive performance [8]. Two meta-analyses of studies on patients with different cancer types and stages reported moderate to large effects of MBIs in reducing symptoms of stress, anxiety and depression [16, 17]. Other studies have demonstrated the efficacy of MBIs to reduce cancer-related chronic fatigue [18, 19] and sleep disturbance [20]; (2) MBIs may improve cognitive performance as some studies have shown that MBIs have the potential to improve attention and working memory by increasing the ability to override irrelevant stimuli [21]. Randomized controlled trials across different populations show improved working-memory capacity [22, 23] and improved executive function [24] in participants who completed MBIs; (3) MBIs may induce recovery of chemotherapy-induced changes in the brain. Studies using brain imaging show effects of mindfulness on white matter regeneration [25] and effects on functional brain connectivity [26] and (4) finally, MBIs can have a positive impact on immune system dynamics [27].

Up till now, one study has examined the effects of mindfulness-based stress reduction (MBSR) on cancer-related cognitive impairment [19]. The authors conducted a randomized clinical trial to test the effects of MBSR vs an active control group (fatigue education and support) in breast and colorectal cancer survivors with moderate to severe fatigue. The authors noted significantly greater improvement on perceived cognitive impairment immediately following the MBSR training and 6 months later, with moderate to large effect sizes. A similar pattern was observed for outcomes assessed by the Stroop Color Word Test [28]. MBSR participants made significantly fewer errors relative to the control group and their accuracy rate increased over time. These effects were small to moderate. More recently, we completed a small proof-of-concept study prior to the here proposed trial [29]. Thirty-three breast cancer patients were randomized to either MBI or a treatment as usual (TAU) control group. Patients in the MBI group showed significant improvement on perceived cognitive impairment compared to the TAU control group with a large within-group effect size in the mindfulness condition (Hedges’ g_av_ .93, 95%CI 3.88–22.16) and no significant effect size in the control condition (Hedges’ g_av_ .12, 95%CI − 1.35–5.87). We also performed resting-state functional magnetic resonance imaging (fMRI) to study changes in brain activation at rest and found an improvement in functional connectivity in the attention network. As these preliminary results are promising we here propose a three-arm, parallel-group, randomized, assessor-blind, controlled clinical trial to investigate the effect of MBI on cognitive impairment after chemotherapy. In this randomized controlled trial (RCT) we will compare MBI to an active physical training condition and a TAU control condition. Impact will be measured using behavioral, psychological and biological outcomes.

## Methods/design

### Aim and objectives

The overall aim of the study is to examine whether MBI can improve cognitive functioning and quality of life for cancer patients after chemotherapy.

The objectives are as follows:

1. Investigate whether MBI improves cognitive functioning in comparison with both control conditions
2. Determine the differences in both structural and functional changes in the brain within (longitudinal) and between the groups; and
3. Explore whether changes in behavioral, psychological and biological outcomes, are associated with improvements in cognitive functioning

Our hypotheses are:

1. Both MBI and the physical training (active control condition) improve cognitive impairment compared to the TAU control condition
2. MBI is more effective than the active control condition for improving cognitive impairment
3. A mechanistic difference will exist between MBI and the active control condition based on brain imaging. More specifically, we expect a stronger impact in the salience and dorsal attention networks, the frontoparietal network and default mode network. These networks have been associated before with aspects of cognitive changes after chemotherapy treatment.

### Design

**Fig. 1:**
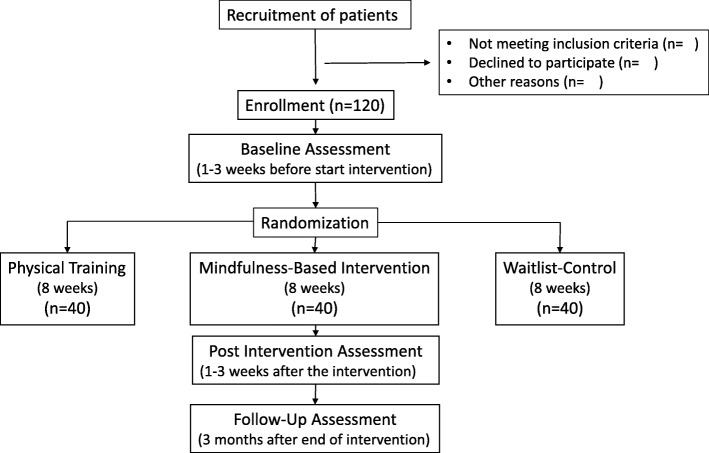
Diagram of planned study flow

**Fig. 2:**
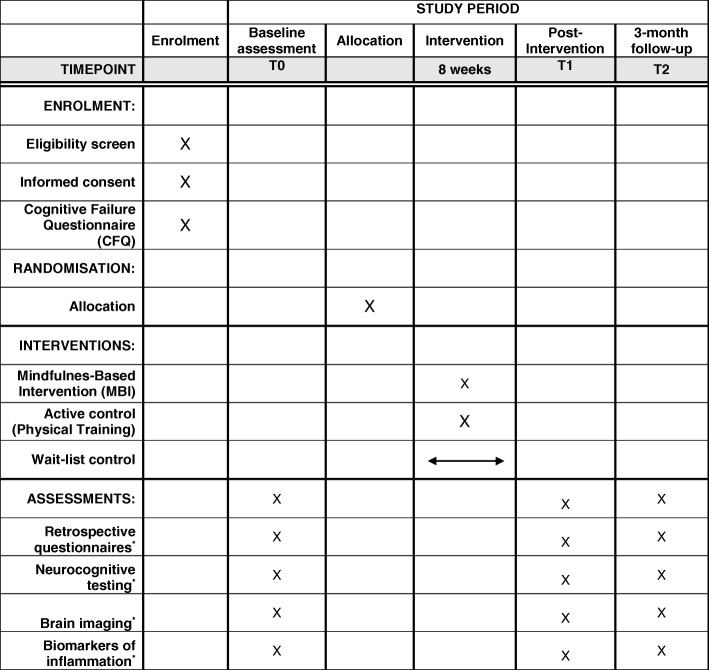
Standard Protocol Items: Recommendations for Interventional Trials (SPIRIT) schedule. *A detailed description of the assessments measures is given in Table 2

This is a single-centre RCT with three study arms and stratified random allocation. Participants in the control condition will receive care from the treatment centre as usual and participants in the intervention groups will receive MBI or a physical training as well as their usual care. The intervention format is equivalent in both intervention conditions. Study evaluation will be done by comparing within and between the three groups. The potential effect will be assessed on a series of outcome measures. Measurements will take place at baseline (i.e., 1–3 weeks before randomization), 1–3 weeks after the intervention and 3 months after the intervention. Participants are allowed to withdraw without giving a reason at any time. There will be no follow-up assessments of these participants. The development of the study protocol followed the SPIRIT (Standard Protocol Items: Recommendations for Interventional Trials) guidelines [30]. The planned flow diagram of this trial is presented in Fig. 1. The protocol is reported according to the Standard Protocol Items: Recommendations for Interventional Trials (SPIRIT; Fig. 2 and Additional file 1). This study was approved by the Medical Ethics Committee of UZ/KU Leuven on 9 July 2018 (S59396). This study was registered with ClinicalTrials.gov (NCT03736460) on 8 November 2018.

### Eligibility criteria

Participants will be included if they had a diagnosis of breast cancer at an early stage with or without solitary metastases (except solitary brain metastases), and completed their treatment (surgery and chemotherapy) a minimum of 6 months and a maximum of 5 years before. Participants have significant cognitive complaints as measured by the Cognitive Failure Questionnaire (CFQ total score > mean study Ponds + 1 standard deviation (SD) or on two or more of the CFQ extra questions (T/E) > mean study Ponds + 1 SD [31];), are between 18 and 65 years old, and have sufficient understanding of Dutch.

Patients with a history of intellectual disability, psychiatric and or neurological disorder will be excluded. Patients will also be excluded if they report a previous participation in a mindfulness-based training or practice meditation regularly.

### Setting and recruitment process

Recruitment will take place in the Multidisciplinary Breast Cancer Centre (MBC), UZ Leuven. Patients will be identified through the outpatient database and study eligibility determined using medical records. Potential candidates will receive a letter with a general outline of the study and will be contacted by phone in order to evaluate their interest. Patients who are interested will be sent the informed consent form and the Cognitive Failure Questionnaire (CFQ). The researcher will follow up by phone to answer any questions related to the study. Only patients with a total score > mean study Ponds + 1 SD will be recruited for the study (see also “Eligibility criteria” above).

### Randomization

Participants are randomized (1:1:1) to the control group or intervention groups stratified by time since chemotherapy completion, age and hormone therapy (Y/N). The randomization will be carried out by an independent researcher using random number lists created in MinimPy, a free, open-source application (http://minimpy.sourceforge.net/). Participants will be randomized by minimization, a covariate-adaptive randomization technique which balances the allocation to groups across specified covariates without compromising randomization [32].

After randomization, participants will be unblinded to group assignment, as the interventions do not allow for blinding. Assessors and statisticians will be blinded.

### Interventions

#### Intervention group – MBI

The MBI adheres to a standardized protocol developed from the MBSR curriculum [33] and the mindfulness-based cognitive therapy for cancer [34]. The program was adjusted for women, who have an active life, combining job activities with (often) motherhood, and consists of four 3-h group sessions spread over 8 weeks. Each session consists of guided experiential mindfulness exercises (e.g., focus on the breath, body scan, breathing space, mindful movement, sitting meditation), sharing of experiences of these exercises, reflection in small groups, psycho-education (e.g., on topics such as stress, fatigue, fear of cancer recurrence, self-care) and review of homework exercises. The program aims to: (1) increase present moment awareness and recognize entanglement with one’s thoughts and emotions; (2) teach acceptance and mindfulness as an alternative strategy for dealing with problematic thoughts and feelings, and how these may be used to facilitate value-based actions. The program is led by two clinical psychologists who are skilled trainers following standardized procedures. In between the group sessions participants are contacted by phone or mail for a short check-up and a kind reminder to continue their daily practice. The training is supported by the use of homework exercises and audio material. Daily home practice will be strongly encouraged.

Attendance to the group sessions will be monitored as well as daily home practice (see further).

The MBI will be delivered by two clinical psychologists with experience in delivering MBIs to breast cancer patients. Treatment fidelity and trainer adherence will be established using randomly selected video-recordings of the sessions that will be analyzed by independent raters. This will be done using the Mindfulness-based Intervention – Teaching Assessment Criteria [35].

#### Active control group – physical training

This intervention is based on the recommended levels of physical activity for adults aged 18–64 years from the World Health Organization. These recommendations are the same for women after any breast cancer treatment [36]. This program will consist of four 2-h group sessions spread over 8 weeks. Each session will consist of psycho-education (e.g., on topics such as the basics of movement, advantages of physical activity, and training principles), endurance and resistance training, stretching, balance and relaxation exercises, sharing of experiences of these exercises and review of homework exercises.

The goal of the program is: (1) to improve physical functioning, physical fitness, strength, flexibility and balance and (2) to increase knowledge about physical activity. Participants will receive exercise material and homework assignments which they are expected to follow daily. The physical training is led by a physiotherapist experienced in oncology revalidation.

Attendance to the group sessions will be monitored as well as daily home practice.

The specific elements of each session in both programs are described in Table 1.

**Table 1:** Content of each session of the mindfulness-based intervention (MBI) and physical training

Program	MBI	Physical training
Session 1	**Discovering that we function on automatic pilot and kind attention to the body** Pause exercise, ground rules, grounding practice, raisin exercise, body scan, sitting meditation 1 (focus on the breath & coming back to it), overview home practices	**Introduction to international physical activity guidelines, responsible training and explanation of the movement diary** Aerobics, strength exercises and stretching. Progressive relaxation. Overview home practices
Session 2	**Kind attention to the body and the breath** Body scan, sitting meditation 2 (focus on the breath and expanding to sensations in the body and the body as a whole), standing in mountain, mindful walking, pleasant experience and entanglement (thoughts – feelings – sensations), 3-min breathing space, “Thought on a thread” practices (feet on the floor, coming to the breath, kind wishes), overview home practices	**Exploring different training principles and the advantages of physical training** Aerobics, Thera-Band strength exercises, stretching. Body scan. Overview home practices
Session 3	**Gently learning to work with personal limits and discovering that we can choose how to respond by opening gently to experience** Mindful movement, coping with fatigue, extended breathing space, unpleasant experience and entanglement (thoughts – feelings – sensations), sitting meditation 3 (focus on the breath and the body, gentle attention to intense, difficult or painful sensations in the body, expanding to sounds, thoughts & feelings, open awareness), “Thought on a thread” practices (feet on the floor, coming to the breath, kind wishes), overview home practices	**Education about healthy movement, injury prevention and spinal hygiene** Step-aerobics, strength exercises with weights, stretching. Guided visualization. Overview home practices
Session 4	**Gently being with what is difficult and taking care of ourselves** Pause, short body scan, mindful movement, sitting meditation 3, “Thought on a thread” practices (feet on the floor, coming to the breath, kind wishes), mindful coping with thoughts, vicious circle of anxious preoccupation, extended breathing space and action of self-care, mountain meditation, review course, overview home practices	**Information about activity trackers and sports watches, tips to continue physical activity** Step-aerobics, strength exercises with Swiss-ball, stretching. Tennis ball massage. Overview home practices

For both programs daily home practice will be assessed using a practice journal made by the instructor. The practice journal has the same format for both intervention conditions with indications for the duration, the frequency and type of exercise. At the end of the 8-week training program, participants hand in the practice journal to the trainer.

#### TAU control group – usual care

Participants in the TAU control group will continue to receive their usual care. They will complete assessments at exactly the same time points as the intervention groups, i.e., at baseline (T0), post intervention (T1) and at 3 -months’ follow-up (T2). Upon completion of the final assessment, the control group will be offered the MBI.

### Measures

#### An overview of measures is given in Table 2. Socio-demographic data

Data to be collected at baseline include age, sex, marital status, education level, professional status.

**Table 2:** Overview of measures and corresponding measurement time points

Measure	Target concept	T0	T1	T2
**Retrospective questionnaires**				
CFQ	Subjective measure of cognitive functioning	x	x	x
DASS	Emotional distress	x	x	x
CIS – subscale severity	Fatigue severity	x	x	x
CHIME	Mindfulness skills	x	x	x
**Neurocognitive testing**				
Bourdon-Wiersma Dot Cancellation Test	Attention and concentration	x	x	x
Auditory Verbal Learning Test	Memory	x	x	x
WAIS III forward digit span	Memory	x	x	x
WAIS III backwards digit span	Executive functioning			
WAIS III letter-number sequencing	Executive functioning	x	x	x
Stroop Color Word Test	Executive functioning	x	x	x
Controlled Oral Word Association Test	Executive functioning	x	x	x
WAIS digit symbol-coding	Cognitive/psychomotor processing speed	x	x	x
9HPT	Cognitive/psychomotor processing speed	x	x	x
TMT – Form A	Cognitive/psychomotor processing speed	x	x	x
TMT – Form B	Attention and concentration/executive functioning			
DART	Verbal IQ	x	x	x
**Brain imaging**				
High-resolution, T1-weighted imaging	Brain structure	x	x	x
Active fMRI during N-back task	Brain activation during N-back task	x	x	x
Resting-state fMRI	Brain connectivity	x	x	x
Diffusion MRI	White matter microstructure	x	x	x
**Biomarkers of inflammation**				
IL-1, IL-6 and TNF-α	Inflammatory process	x	x	x

Abbreviations: *T0* baseline, *T1* 1–3 weeks post intervention*, T2* 3 months post intervention*, 9HPT* Nine-hole Peg Test, *CFQ* Cognitive Failure Questionnaire, *CHIME* Comprehensive Inventory of Mindfulness Experiences, *CIS* Checklist Individual Strength, *DART* Dutch Adult Reading Test, *DASS* Depression, Anxiety, Stress Scales, *fMRI* functional magnetic resonance imaging, *IL* interleukin, *MRI* magnetic resonance imaging, *TMT* Trail Making Test, *TNF* tumor necrosis factor, *WAIS* Wechsler Adult Intelligence Test

#### Retrospective questionnaires

##### Cognitive Failure Questionnaire

The Cognitive Failure Questionnaire (CFQ) is used to obtain information on subjective cognitive function [37]. The CFQ consists of 25 items assessing self-reported cognitive failures in daily activities, such as forgetting what the person went into a room to do. Questions are rated on a 5-point scale ranging from 0 = “never” to 5 = “very often.” Subscales on distraction, distraction in social situations, names and word-finding, orientation, and a total summary score are available. Four extra questions assess whether symptoms increased over the past 5 years. The scale has shown high internal consistency and good construct and criterion validity in groups of adult patients with cancer [10, 38].

##### Emotional distress

Emotional distress is measured using the Depression Anxiety Stress Scales (DASS-21) [39]. The DASS-21 consists of three 7-item scales designed to assess depression (DASS-21-D), anxiety (DASS-21-A) and stress symptoms (DASS-21-S). The total scale score is used as a measure of general distress. The scale is a valid and reliable measure for use among cancer patients [40].

##### Fatigue

Fatigue is measured with the fatigue severity subscale of the Checklist Individual Strength (CIS) [41]. This subscale consists of eight items, each scored on a 7-point Likert scale, with higher scores reflecting more fatigue. The CIS has shown high internal consistency and good construct and criterion validity in a group of adults with different cancer types [18].

##### Mindfulness skills

The 37-item Comprehensive Inventory of Mindfulness Experiences (CHIME) is used to measure mindfulness [42]. Items are rated on a 6-point scale ranging from 1 to 6, with higher scores indicating higher levels of mindfulness. The CHIME provides eight subscales: awareness of internal experiences, awareness of external experiences, acting with awareness, accepting and non-judgmental orientation, decentering and non-reactivity, openness to experiences, relativity of thoughts, and insightful understanding. In the present study the total score and scores of the subscales will be used. The CHIME is a valid and reliable measure for use among adults [43, 44].

##### Health-related quality of life

Health-related quality of life will be assessed using the Quality of Life Questionnaire (QLQ-C30) [45]. This is a multidimensional, cancer-specific quality-of-life questionnaire developed by the European Organization for Research and Treatment of Cancer (EORTC) Study Group on Quality of Life for use in international clinical trial settings. It includes five functional scales (physical, role, emotional, social and cognitive functioning), three symptom scales (fatigue, pain, nausea and vomiting) and a global health status/quality of life scale. In this study we will only use the global health status/quality of life scale.

### Neurocognitive tests

Objective cognitive performance is evaluated using a neurocognitive test battery, covering several domains: (1) Attention and concentration (Bourdon-Wiersma Dot Cancellation Test [45, 46], Trail Making Test (TMT) [46–49]); (2) Memory (Auditory Verbal Learning Test (AVLT), parts A and B [46], Weschler Adult Intelligence Scale (WAIS) III forward digit span [50]); (3) executive functioning (Stroop Color Word Test [51, 52], Controlled Oral Word Association Test (COWAT) [46, 53], Trail Making Test (TMT), form B [46–49], WAIS III backward digit span, and WAIS III letter-number sequencing [50]) and (4) cognitive/psychomotor processing speed (WAIS III digit symbol-coding [50], Nine-hole Peg Test (9HPT) [54, 55] and Trail Making Test (TMT), form A [46–49]). Additionally, verbal IQ is estimated by the Dutch Adult Reading Test (DART) [56]. The neuropsychological test battery has high reliability and good validity in our study population [9–11].

### Structural and functional changes in the brain

Non-invasive MRI imaging of the brain (high-resolution, anatomical, T1-weighted imaging, (T1-w imaging) multi-shell diffusion imaging (DWI) and functional MRI (fMRI) will be used to study both structural and functional changes in the brain. All subjects are imaged on a 3-Tesla scanner (Achieva, Philips, Amsterdam, the Netherlands) with a 32-channel phased-array head coil.

#### High-resolution, 3D, T1-weighted image (3D-T1-w, duration ± 5 min)

The 3D-T1-w anatomical image will be acquired using a 3D-turbo field echo (TFE) sequence and is used to assess volumetric changes in the brain and as anatomical reference for both functional and diffusion MRI.

#### Resting-state functional MRI (rs-fMRI, duration ± 7 min)

Whole-brain, T2*-weighted echo planar images (EPIs), sensitive to blood oxygenation level dependent (BOLD) contrast will be used to acquire functional MRI (fMRI) scans during “resting state.” Participants are asked to close their eyes, lie still and not to fall asleep.

#### Active functional MRI (afMRI, duration ± 8 min)

Whole-brain, T2*-weighted EPIs, sensitive to blood oxygenation level dependent (BOLD) contrast will be used to acquire functional MRI (fMRI) scans while participants perform a memory task in the scanner. The fMRI task consists of a visual N-back sequential letter task used to assess working memory brain activation [46, 47]. Four conditions are tested: 0-back, 1-back, 2-back and 3-back in a blocked design. The 0-back control condition has a minimal working-memory load; participants need to decide if the current letter matches a single target letter that was specified before. In the 1-back condition, participants need to asses if the current letter matches the previous letter. During the 2-back (3-back) condition, participants need to assess whether the current letter matches the letter that has been presented 2-back (3-back) in the sequence. Participants practice the task before the scanning session. During the MRI scanning session, participants respond by pressing a button to indicate whether the item matches the target condition.

#### Diffusion-weighted imaging (DWI, duration ± 20-30 min)

Whole-brain, multi-shell diffusion-weighted EPIs will be acquired with low and high b values, ranging from 0 to 4000 and with number of directions ranging from 20 to 60. Diffusion images will be used to assess microscopic white matter microstructural differences.

### Biomarkers of inflammation

Blood samples will be collected from all participants at the three time points, on the same day as completion of the other assessments. Levels of pro-inflammatory cytokines such as interleukin (IL)-1, IL-6, IL-8, tumor necrosis factor (TNF)-α, interferon (IFN)-γ and monocyte chemoattractant protein-1 (MCP-1) and C-reactive protein (CRP) will be determined with bead-based multiplex immunoassays and compared between groups and time points.

### Analysis plan

Primary outcome measures:

1. The difference in mean change scores from the baseline on the CFQ between groups; and
2. The difference in mean change scores from the baseline on the connectivity in the attention network between groups.

Secondary outcome measures:

1. Changes in neurocognitive test scores
2. Changes in emotional distress, fatigue and mindfulness skills
3. Changes in brain white and gray matter structure
4. Changes in functional brain activity during N-back task
5. Changes in functional connectivity during resting state; and
6. Changes in biomarkers of inflammation

### Data analysis

Analyses will be done both per protocol and intention to treat. Analyses are based on general linear modelling and multilevel mixed-effects modelling. The impact of the intervention will be tested (confirmatory) via a multilevel model with two levels (time points nested within persons). Results will be controlled for false discovery rate [48]. Predictors are time (level-1 predictor), condition (level-2 predictor) and cross-level interactions. To evaluate the process of change a mediation analysis will be performed. Mediation will be tested (exploratory) by adding potential mediators (e.g., different mindfulness facets) and their interaction with condition and time in the intervention model. Time practicing will be tested (exploratory) as a potential moderator. Models are based on the procedure described in Bauer et al. (2006) [49].

State of the art image processing techniques will be used to analyze the MRI images and study both structural and functional differences longitudinally within the groups and between the groups. Voxel-based morphometry (VBM) will be used to study structural changes in the brain. Additionally, advanced multi-shell diffusion images will be processed and analyzed using ExploreDTI/Metrics (DTI, fixel-based analysis, CSD tractography), NODDI modelling software and in-house developed software. This will include head-motion and eddy-current correction and will use inverse phase encoding images and B0 maps for optimizing correction procedures. Seed-based analysis, independent component analysis (ICA) and graph theory will be applied to analyze the resting state fMRI data and assess brain connectivity. From the MRI data we will generate maps reflecting grey matter volume, white matter properties (e.g., fractional anisotropy, neurite density index, orientation dispersion index) and brain connectivity. Voxel-based statistical analysis using the general linear model and non-parametric statistics will be used to find significant differences (*p* < 0.05) in imaging parameter maps between time points and groups. Statistical parametric mapping on a voxel-by-voxel basis will be conducted by using a general linear model approach to assess brain activation during the N-back memory task. Contrast images comparing pairs of working-memory load conditions (e.g., 3-back > 0-back) will be created for each patient and will be used in second-level analyses to assess differences between time points and groups (confirmatory tests for H1 and H2, exploratory test for H3).

The association between the obtained outcomes, psychological and behavioral outcomes based on the scores of retrospective questionnaires, neuroimaging parameter maps, the performance on neurocognitive tests and biomarkers of inflammation will be investigated using correlation analysis.

The number of patients in this study (40 patients in each group) is based on: (1) earlier studies that investigated the effects of MBI on the brain in which effects where shown after MBI in groups of *n* = 20 (longitudinal design [50] and *n* = 13 (cross-sectional design) [51, 52]; (2) the one study that already investigated the impact of MBI on cognitive impairment after cancer treatment. This study reported an impact of MBI on cognitive functioning with a design of *n* < 40 in the different study arms [53].

### Data management

Data are collected by the research staff and will be stored in a database in an in-house protected server at KU Leuven. Confidentiality of participants’ data is ensured by using participants’ IDs rather than identifiable information in the data set (i.e., coding) and by storing the document linking the IDs to the identifiable information separately and securely. Only researchers directly involved in the analysis of the RCT will have access to the final trial data set, which will only contain coded data. After data collection and before data storage, all outcomes are manually double-checked by the research staff. The safety, progress, study integrity and design aspects will be monitored at various meetings by the research team involved in this study.

## Discussion

Based on the preliminary results of one randomized controlled trial [53] and a small-scaled pilot study conducted by our research group [29], it is expected that MBIs may have the potential to reduce cognitive impairment after chemotherapy. MBIs could influence and improve cognitive functioning through different pathways, by (1) alleviating symptoms of depression, anxiety and stress; (2) reducing cancer-related chronic fatigue; (3) inducing recovery of chemotherapy-induced changes in the brain; and (4) changing immune system dynamics. This larger scale study, including an active control condition based on physical training, should provide information on intervention-specific effects assessed by psychological, behavioral and biological parameters characteristic for the four different pathways. Combined with an investigation of possible mediators, this study should contribute to basic science-unravelling mechanisms of change underlying the effects of MBI on cognitive impairment and well-being in general.

This study is particularly important not only because of the heavy burden of cognitive impairment following chemotherapy but also because of the great need and demand from patients to pay special attention to these symptoms during revalidation. Therefore, finding a low-threshold therapeutic intervention that can help to relieve cancer-related cognitive impairment can be of major importance. If the study shows that MBI has positive effects on cognitive outcomes in breast cancer survivors, MBI can be made available on a larger scale, for instance by integrating it into the standard revalidation program. The MBI format (four group sessions with in-between online support and feedback of the trainer) as tested in this study is an ideal format to implement it in the existing revalidation programs.

## Trial status

The protocol number is S59396 and concerns version 5 (dd. 1 June 2018). Recruitment started in September 2018 and will approximately be finished in February 2020.

## Supplementary information

**Additional file 1.** Standard Protocol Items: Recommendations for Interventional Trials (SPIRIT) Checklist.

## Data Availability

The data set generated and analyzed during the current study will be available from the corresponding author on reasonable request.

## Abbreviations

9HPT: Nine-hole Peg Test
afMRI: Active functional magnetic resonance imaging
AVLT: Auditory Verbal Learning Test
BOLD: Blood oxygenation level dependent
CFQ: Cognitive Failure Questionnaire
CHIME: Comprehensive Inventory of Mindfulness Experiences
CIS: Checklist Individual Strength
COWAT: Controlled Oral Word Association Test
CRP: C-reactive protein
DART: Dutch Adult Reading Test
DASS-21: Depression Anxiety Stress Scales
DWI: Diffusion-weighted imaging
EORTC: European Organization for Research and Treatment of Cancer
EPI: Echo planar image
fMRI: Functional magnetic resonance imaging
ICA: Independent component analysis
IFN: Interferon
IL: Interleukin
MBC: Multidisciplinary Breast Cancer Centre
MBI: Mindfulness-based intervention
MBSR: Mindfulness-based stress reduction
MCP-1: Monocyte chemoattractant protein-1
QLQ-C30: Quality of Life Questionnaire
RCT: Randomized controlled trial
rs-fMRI: Resting-state functional magnetic resonance imaging
SD: Standard deviation
SPIRIT: Standard Protocol Items: Recommendations for Interventional Trials
T1-w imaging: T1-weighted imaging
T2*-weighting highlights differences in the T2 relaxation time of tissues: It is a pulse sequence in MRI and captures the effect of external factors on the T2 relaxation time
TAU: Treatment as usual
TFE: Turbo field echo
TMT: Trail Making Test
TNF: Tumor necrosis factor
VBM: Voxel-based morphometry
WAIS III: Wechsler Adult Intelligence Scale
WM: White matter
